# Genomic investigation and nationwide tracking of pediatric invasive nontyphoidal *Salmonella* in China

**DOI:** 10.1002/mlf2.12117

**Published:** 2024-03-29

**Authors:** Yefang Ke, Lin Teng, Zhe Zhu, Wenbo Lu, Wenyuan Liu, Haiyang Zhou, Qi Yu, Lina Ye, Pan Zhu, Guoping Zhao, Min Yue

**Affiliations:** ^1^ Department of Clinical Laboratory Ningbo Women and Children's Hospital Ningbo China; ^2^ Department of Veterinary Medicine Zhejiang University College of Animal Sciences Hangzhou China; ^3^ Department of Blood Transfusion Ningbo No. 2 Hospital Ningbo China; ^4^ Office of Screening Ningbo Women and Children's Hospital Ningbo China; ^5^ Neonatal Intensive Care Unit Ningbo Women and Children's Hospital Ningbo China; ^6^ School of Life Science, Hangzhou Institute for Advanced Study University of Chinese Academy of Sciences Hangzhou China; ^7^ CAS Key Laboratory of Synthetic Biology, Institute of Plant Physiology and Ecology, Shanghai Institutes for Biological Sciences Chinese Academy of Sciences Shanghai China; ^8^ Department of Microbiology and Microbial Engineering, School of Life Sciences Fudan University Shanghai China; ^9^ State Key Laboratory for Diagnosis and Treatment of Infectious Diseases, National Clinical Research Center for Infectious Diseases, National Medical Center for Infectious Diseases, The First Affiliated Hospital, College of Medicine Zhejiang University Hangzhou China; ^10^ Hainan Institute of Zhejiang University Sanya China

## Abstract

Invasive nontyphoidal *Salmonella* (iNTS) causes significant concern with ~15% morbidity, affecting populations mainly in African countries. However, iNTS infections among the Chinese pediatric population remain largely unknown. Here, we conducted a genomic investigation to study pediatric iNTS infections in a Chinese hospital. iNTS isolates accounted for 15.2% (18/119) of all nontyphoidal *Salmonella* (NTS) strains. Compared to non‐iNTS isolates, iNTS isolates harbored a lower prevalence of antimicrobial‐resistant genes of fluoroquinolones and β‐lactams, as well as disinfectant determinants and plasmids, but carried a significantly higher prevalence of *cdtB*, *faeCDE*, and *tcpC* genes. Importantly, we detected an emerging serovar Goldcoast as the predominant iNTS serovar locally. By integrating 320 global Goldcoast genomes based on the One Health samplings, we conducted nationwide phylogenomic tracking and detected repeated human‐to‐human transmission events among iNTS cases caused by an underestimated serovar Goldcoast. Together, our exploratory genomic approach highlights a new trend in pediatric iNTS infections.

Nontyphoidal *Salmonella* (NTS) typically causes self‐limiting diarrheal illnesses^1^. However, NTS infection can also present as an invasive febrile disease with bacteremia, meningitis, or focal infections, with a high case fatality rate of ~15%^2^. Children are susceptible to acquiring invasive NTS (iNTS) disease^2^. Previous studies were mainly focused on the serovar and antimicrobial‐resistant phenotype of iNTS isolates; the genomic characteristics of pediatric iNTS infection and possible sources for iNTS transmission remain largely unknown in China^3^, ^4^, ^5^. Herein, we conducted a cutting‐edge investigation with whole‐genome sequencing (WGS) to understand iNTS in China and suggest potential sources for an emerging iNTS serovar.

From August 2020 to November 2022, a total of 119 NTS isolates were identified from children in Ningbo Women and Children's Hospital (Table S1). A total of 101 NTS isolates were isolated from stool samples and defined as noninvasive isolates, while 17 iNTS isolates were isolated from blood, and one iNTS isolate recovered from a perianal abscess was defined as an invasive isolate. The iNTS and non‐iNTS infection cases showed similar characteristics on baseline demographics, symptoms, and laboratory examination results, except that non‐iNTS infection cases had a higher proportion of gastrointestinal symptoms—diarrhea and mucus in stools (*p* < 0.05) (Table S2).

A previous study suggested that iNTS infection rarely leads to death in China^5^, and all of the 18 children in this study were recovered after invasive infections. In contrast, iNTS infections are always linked with high mortality rates in other countries. We suspect this distinction might be related to several reasons. First, the virulence of epidemic iNTS isolates may differ between different regions. Second, the relatively high drug‐resistant NTS isolates in some places may delay clinical treatment, such as the multidrug‐resistant *Salmonella* Typhimurium ST313 in Africa, while the low resistance of iNTS isolates in this study may render more effective and timely treatment^6^. Finally, the patients in this study were in relatively good condition. Nevertheless, clinical investigations with a broad population in different regions of China are highly warranted.

WGS was performed to identify the genomic hallmarks of iNTS isolates by comparing with non‐iNTS isolates, which showed that iNTS and non‐iNTS isolates had different patterns of antimicrobial resistance (AMR) and virulence. Through phenotypic and genomic analyses, we noticed the local iNTS isolates were less resistant, which confirmed our previous findings from 2012 to 2019^4^. The phenotypic and genotypic resistance had an overall concordance of 92.2%, with sensitivity and specificity of 90.4% and 93.0%, respectively (Tables S3 and S4). First, from phenotypic analysis, iNTS isolates had significantly lower resistance rates to Levofloxacin, Ciprofloxacin, Aztreonam, Cefepime, Ceftriaxone, Trimethoprim‐sulfamethoxazole (TMP‐SMX), Ampicillin/sulbactam, and Ampicillin (*p* < 0.05). Most (83.3%, 15/18) of iNTS isolates were susceptible to all the antimicrobial agents tested (Figure 1A). Then, in the view of genomics, the average number of antimicrobial resistance genes (ARGs) in iNTS isolates (median: 1, interquartile range [IQR]: 1–2.25) was significantly lower than that in non‐iNTS isolates (median: 9, IQR: 6–12) (*p* < 0.001). Specifically, the prevalence of iNTS isolates, which carried at least one fluoroquinolone resistance determinant, β‐lactamase genes, and *tet* genes, was less than that of non‐iNTS isolates (5.6%, 1/18 vs. 65.3%, 66/101, *p* < 0.001; 22.2%, 4/18 vs. 79.2%, 80/101, *p* < 0.001; and 22.2%, 4/18 vs. 71.3%, 72/101, *p* < 0.001) (Figure 1B). In addition, the lower rate of multi‐drug resistance (MDR) isolates (Figure 1C,D), and disinfectant resistance genes (Figure 1E), and fewer harboring plasmids correlated with lower AMR in iNTS isolates (Figure S1).

**Figure 1:**
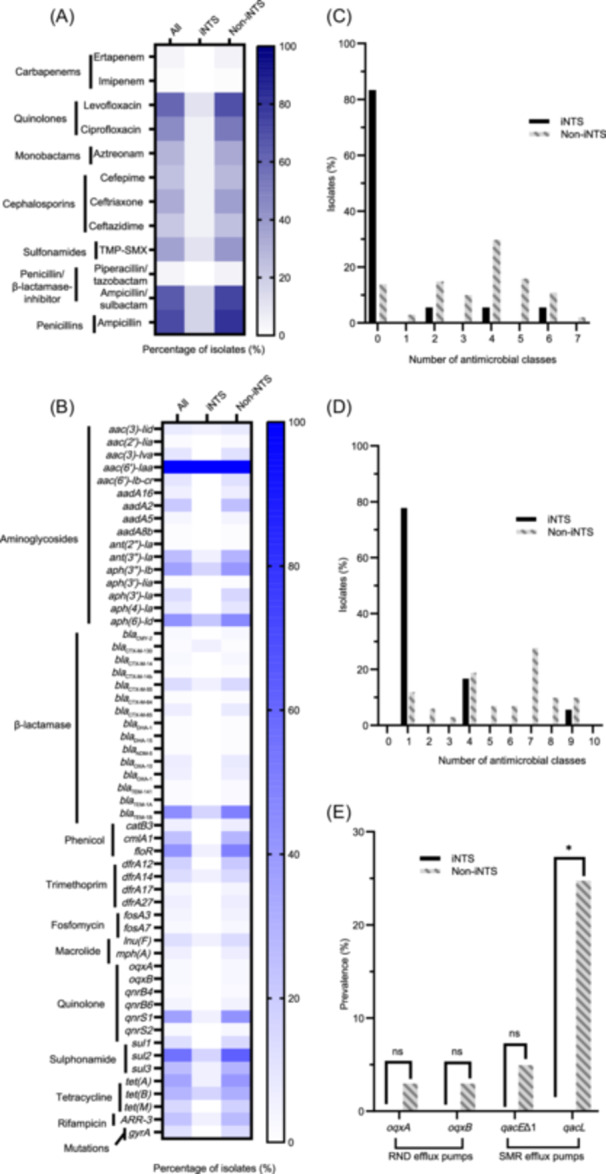
Phenotypic and genotypic AMR between invasive non‐typhoidal *Salmonella* (iNTS) and non‐iNTS groups. (A) Percentage of isolates with phenotypic resistance to distinct antimicrobials. (B) Percentage of isolates with AMR determinants. (C) Distribution of isolates with phenotypic resistance towards different antimicrobial classes. (D) Distribution of isolates with different classes of AMR determinants. (E) Prevalence of disinfectant resistance genes between the iNTS and NTS groups. RND efflux pump and SMR efflux pump were found. A chi‐squared test was conducted. *p < 0.05; ns, no significant difference. AMR, antimicrobial resistance; RND, reresistance‐nodulation‐division; SMR, small multidrug resistance; TMP‐SMX, Trimethoprim‐sulfamethoxazole.

The third‐generation Cephalosporin and Ciprofloxacin have now been recommended as first‐line drugs to treat severe and invasive NTS infections^6^. A previous study^7^ has shown that the resistance rates to third‐generation Cephalosporin and Ciprofloxacin are relatively high among iNTS isolates in China. In our study, the resistance rates of iNTS isolates to Ceftriaxone (5.6%) and Ciprofloxacin (5.6%) were all lower than the above rates; this might be because most of the iNTS isolates (83.3%) exhibited pan‐susceptibility, which renders an effective treatment. However, as the sample size is relatively small, continued surveillance of sensitivity to the third‐generation Cephalosporin and Ciprofloxacin is needed.

On the other hand, there was a difference between the two groups (iNTS vs non‐iNTS) regarding virulence genes (Figure S2). The prevalence of *cdtB*, *faeCDE*, and *tcpC* was significantly higher, while the prevalence of *lpfABCDE*, *sodCI*, *ratB*, *gogB*, *ssel/srfH*, *sspH2*, and *pipB2* was considerably lower among iNTS isolates than non‐iNTS isolates (*p* < 0.05). Intriguingly, most of the iNTS isolates (72.2%, 13/18) clustered together. All of these clustered iNTS isolates had virulence factors of *cdtB*, but none carried *lpf*, *sodCI*, *gogB*, *ssel/srfH*, and *sspH2*. The *cdtB gene* encodes typhoid toxin, which leads to G2/M‐phase growth arrest of the target cell and, ultimately, cell death^8^. Besides, it has been found that the pseudogenes of *ratB* and *ssel* were involved in the invasion of *S*. Typhimurium ST313^9^, ^11^. The *ratB* gene was associated with intestinal persistence in a murine model, and the *sseI* gene encoding an effector inhibits dendritic cell (DC) migration^10^, ^11^. The differences in virulence genes between iNTS and non‐iNTS isolates may involve different infection outcomes.

In silico analysis showed 25 defined serovars (Figure S3A), and serovar 1,4,[5],12:i:‐ was sampled predominantly in non‐iNTS isolates (42.6%, 43/101) (Figure S3B). Goldcoast was the most common serovar in iNTS isolates (22.2%, 4/18), followed by 1,4,[5],12:i:‐ (16.7%, 3/18). The other iNTS serovars included Saintpaul (*n* = 2), Oranienburg (*n* = 2), Muenster (*n* = 1), Give (*n* = 1), Poona (*n* = 1), Johannesburg (*n* = 1), Rissen (*n* = 1), Javiana (*n* = 1), and Bovismorbificans (*n* = 1) (Figure S3C).

Foodborne outbreak cases of *Salmonella* Goldcoast were mainly reported in Europe before^12^, ^13^, ^14^, ^15^; however, they have been increasingly observed in both the Chinese mainland and Taiwan, China nowadays^16^, ^17^. Considering an emerging *Salmonella* serovar associated with human infections in China, One Health scale genomic tracking for *S*. Goldcoast was performed. Seven local *S*. Goldcoast isolates (four iNTS and three non‐iNTS isolates) from the present study, contextualized with 320 global isolates, were included in the phylogenomic analyses. The maximum‐likelihood phylogenetic tree based on core‐genome SNPs revealed that these isolates formed four major lineages (L‐I, L‐II, L‐III, and L‐IV). L‐I, L‐III, and L‐IV comprised isolates mostly from China, while L‐II mainly consisted of isolates from other regions (Figure S4).

Most of the *S*. Goldcoast isolates in the present study (71.4%, 5/7) belonged to L‐IV, including three invasive isolates (NBFE‐57, NBFE‐129, and NBFE‐138) and two noninvasive isolates (NBFE‐77 and NBFE‐119). NBFE‐57, NBFE‐129, NBFE‐138, and NBFE‐77 isolates were closely related to several isolates recovered from human stool samples in Shanghai, China, during 2014–2016, within a distance of 15 SNPs. Moreover, the NBFE‐77 isolate was also closely related to an isolate (SAL00855) recovered from the water sample in Zhejiang, China in 2015, while NBFE‐129 and NBFE‐138 isolates were both closely related to an isolate (IF2694) recovered from the soil sample in Jiangsu, China in 2021. The other two isolates belong to L‐I (NBFE‐41, an invasive isolate) and L‐III (NBFE‐125, a noninvasive isolate). NBFE‐41 isolate, which was recovered from a boy in 2021, was closely related to the 1096_ZJNB22SAL200 isolate recovered from a 65‐year‐old female in 2022, with only one SNP difference. Both of them were isolated from blood samples, and their hosts lived in two geographically close districts of Ningbo City. Notably, NBFE‐125 isolate, which was isolated from a stool sample of a girl aged 11 months, was closely related to the isolates recovered from human stool samples in Taiwan, China in 2018 (i.e., isolates 29351, R18.0450, R18.0411, and Sal‐5767), as well as the isolates recovered from a female's stool sample in Zhejiang province, 2020 (Supporting Information: Dataset S1).

Based on the phylogenomic analyses, six of the seven isolates (except for NBFE‐119) in this study were closely related to the isolates recovered from the southeast coastal areas of the Chinese mainland and Taiwan, China. Contaminated pork products were considered the primary sources of the *S*. Goldcoast outbreak reported in Germany^15^, Italy^12^, and Hungary^14^, and whelk was responsible for the outbreak of *S*. Goldcoast in England^13^. As Ningbo is a coastal city in Eastern China, we proposed that meat or seafood might be an important vehicle for the local *S*. Goldcoast circulation.

More and more evidence supports human‐to‐human transmission as a pathway of iNTS infection^18^. Post et al.^19^ surveyed 32 indexes of iNTS‐infected children with their households, livestock, and water samples in Burkina Faso. Three pairs of index patients and their households were identified by Multilocus variable‐number tandem‐repeat analysis (MLVA) (identical or similar MLVA type) and WGS analyses (household isolates have 0–2 SNPs from the index isolates), but we did not find matched pairs from livestock and water samples. Similarly, in Malawi, Koolman et al.^20^ observed two pairs of index cases and their asymptomatic household members from 60 index cases, but they did not detect overlaps between iNTS isolates and animal or environmental isolates. In this study, four invasive *S*. Goldcoast isolates were all found to be closely related to several bacteria of human origin (<15 SNPs), suggesting a potential human‐to‐human transmission mode. However, up to now, the source causing iNTS disease is still unclear in China, and relative studies are strongly encouraged.

For a better interpretation of our findings, some limitations should be considered. (1) This study was a snapshot of the overall genomic characteristics of local pediatric iNTS isolates in a children's hospital. Considering the diversity of *Salmonella* serovars in different places, future studies should recruit multi‐centers with a large sample size. (2) Although we observed differences in virulence genes based on the infection model, their significance remains unknown and needs further experiments to be determined. (3) Since current Goldcoast cases were compared with the available historical collection, rational studies on the households, livestock, environment, and food of iNTS patients are needed to understand the potential transmission route. Nonetheless, our WGS approach demonstrated that iNTS isolates had lower AMR rates than non‐iNTS isolates among pediatric patients in one city in Eastern China. Moreover, iNTS and non‐iNTS isolates showed different virulence factor patterns. With an integrated genomic approach and newly found iNTS serovars, this study highlights a new trend in pediatric Salmonellosis and iNTS infections in China.

## AUTHOR CONTRIBUTIONS

**Yefang Ke**: Conceptualization (equal); investigation (equal); methodology (equal); project administration (equal); resources (equal); writing—original draft (equal). **Lin Teng**: Investigation (equal); resources (equal); software (equal); visualization (equal). **Zhe Zhu**: Investigation (equal); resources (equal). **Wenbo Lu**: Methodology (equal). **Wenyuan Liu**: Investigation (equal); methodology (equal). **Haiyang Zhou**: Methodology (equal). **Qi Yu**: Investigation (equal). **Lina Ye**: Methodology (equal). **Pan Zhu**: Methodology (equal). **Guoping Zhao**: Writing—review and editing (equal). **Min Yue**: Conceptualization (equal); data curation (equal); resources (equal); supervision (equal); writing—review and editing (equal).

## ETHICS STATEMENT

The ethics committee of Ningbo Women and Children's Hospital approved this study (EC2022‐022).

## CONFLICT OF INTERESTS

The authors declare no conflict of interests.

## Supporting information

Supporting information.

Supporting information.

Supporting information.

## Data Availability

All the sequencing data of the *Salmonella* isolates from this study are deposited in the NCBI database under BioProject number PRJNA998333.
