# Supplementary material for: Genomic investigation and nationwide tracking of pediatric invasive nontyphoidal Salmonella in China
Source: mLife. 2024 Mar 29;3(1):156–60. doi: 10.1002/mlf2.12117 (PMC11139200; doi:10.1002/mlf2.12117)
Supplement: Supplementary file 1 — Supporting information. [file MLF2-3-156-s001.docx]

**Supporting Information**

**Genomic investigation and nationwide tracking of pediatric invasive non-typhoidal *Salmonella* in China**

Yefang Ke^1a^, Lin Teng^2a^, Zhe Zhu^3a^, Wenbo Lu^1^, Wenyuan Liu^1^, Haiyang Zhou^2^, Qi Yu^4^, Lina Ye^1^, Pan Zhu^5^, Guoping Zhao^6,7,8^, Min Yue^2,6,9,10 *^

^1^ Department of Clinical Laboratory, Ningbo Women and Children’s Hospital, Ningbo, China;

^2^ Department of Veterinary Medicine, Zhejiang University College of Animal Sciences, Hangzhou, China;

^3^ Department of Blood Transfusion, Ningbo No.2 Hospital, Ningbo, China;

^4^ Office of Screening, Ningbo Women and Children’s Hospital, Ningbo, China;

^5^ Neonatal Intensive Care Unit, Ningbo Women and Children’s Hospital, Ningbo, China;

^6^ School of Life Science, Hangzhou Institute for Advanced Study, University of Chinese Academy of Sciences, Hangzhou, China;

^7^ CAS Key Laboratory of Synthetic Biology, Institute of Plant Physiology and Ecology, Shanghai Institutes for Biological Sciences, Chinese Academy of Sciences, Shanghai, China;

^8^ Department of Microbiology and Microbial Engineering, School of Life Sciences, Fudan University, Shanghai, China;

^9^ State Key Laboratory for Diagnosis and Treatment of Infectious Diseases, National Clinical Research Center for Infectious Diseases, National Medical Center for Infectious Diseases, The First Affiliated Hospital, College of Medicine, Zhejiang University, Hangzhou, China;

^10^ Hainan Institute of Zhejiang University, Sanya, China.

^a^ Yefang Ke, Lin Teng and Zhe Zhu contributed equally to this work.

Correspondence: Min Yue, Department of Veterinary Medicine & Institute of Preventive Veterinary Sciences, Zhejiang University College of Animal Sciences, Hangzhou, Zhejiang, China (myue@zju.edu.cn)

**Materials and Methods**

**Patient information and recovered bacterial isolates**

NTS strains were collected from children aged below 14 years who were admitted to Ningbo Women and Children’s Hospital between August 2020 and November 2022. Cases with NTS infections were divided into iNTS and non-iNTS based on different infection sites. A case of iNTS infection was defined as NTS strains isolated from blood samples or other normally sterile sites. Accordingly, these isolates were characterized as iNTS isolates. A case of non-iNTS infection was defined as NTS strains that recovered from samples of other sites, e.g., stools, and these isolates were defined as non-iNTS isolates. Demographics, clinical data of cases with NTS infection, and information on microorganisms were collected from their electronic medical records.

**NTS identification and antimicrobial susceptibility testing**

The standardized indications for sampling, bacterial culture operations, and NTS identification were performed according to the previously described methods ^1^. Briefly, routine blood cultures were performed on every patient on admission and sampled again when they had signs of systemic infection; then, the blood samples were cultured in the BACT/ALERT 3D system (BioMérieux, France). The stools were sampled after gastrointestinal symptoms and then incubated on *Salmonella*-*Shigella* agar plates. Other samples were collected from related sites when they showed local infection signs and were incubated on blood agar plates. The suspected colonies were picked and identified by biochemical testing and confirmed by matrix-assisted laser desorption ionization-time of flight mass spectrometry (MALDI-TOF MS) (BioMérieux, France).

The antibiotic susceptibility testing of NTS was performed using the VITEK 2 COMPACT automatic analysis system (BioMérieux, France) employing an AST GN13 panel. *Escherichia coli* ATCC25922 was used as a microbiological quality control. The susceptibilities to twelve antimicrobials, including Ertapenem, Imipenem, Levofloxacin, Ciprofloxacin, Aztreonam, Cefepime, Ceftriaxone, Ceftazidime, Trimethoprim-sulfamethoxazole, Piperacillin/tazobactam, Ampicillin/sulbactam, and Ampicillin, were assessed. The antimicrobial susceptibility breakpoints were interpreted according to the Clinical and Laboratory Standards Institute guidelines (CLSI-2022). As the AST GN13 panel has a detection limit of Ciprofloxacin (0.25 μg/mL), intermediate inhibitory concentrations of Ciprofloxacin could not be evaluated below 0.25 μg/mL. For ease of data interpretation, AMR was defined as intermediate (decrease in susceptibility) or resistant minimum inhibitory concentration (MIC), and those who showed resistance to three or more antimicrobial classes were classified as MDR strains. First-line treatment agents included the following: Ampicillin, Ceftriaxone, Ciprofloxacin, and Trimethoprim-sulfamethoxazole.

**Whole genome sequencing and bioinformatic analysis**

Genomic DNA of NTS isolates was extracted using a TIANamp Bacteria DNA kit (Tiangen Biotech, China) and quantified using the Qubit 3.0 Fluorometer (Invitrogen, CA, US). Afterwards, a genomic DNA library was prepared using a NovaSeq XT DNA library construction kit (Illumina, USA), followed by sequencing on the Illumina NovaSeq 6000 Platform. The raw sequence reads were checked for quality and assembled using SPAdes v3.13.1. Then, the *in silico* serotyping was predicted by using the *Salmonella In Silico* Typing Resource (SISTR) (<https://github.com/phac-nml/sistr_cmd>), and virulence genes were analyzed based on the virulence factors database (VFDB). The sequence types, antimicrobial resistance genes (ARGs), disinfectant resistance genes, and plasmid replicons were detected using the assemblies of the samples on the in-house Galaxy platform in combination with MLST v2.22.0 (<https://github.com/tseemann/mlst>), and Abricate v1.0.1 (<https://github.com/tseemann/abricate>), including ResFinder database, MEGARes database, and PlasmidFinder database, all with an 80% identify threshold and an 80% minimum coverage length. The genomic mutations conferring the quinolone resistance-determining region (QRDR) were detected by using Staramr software against PointFinder v1.9 (<https://github.com/phac-nml/staramr>).

**Phylogenomic analysis**

The core-genome SNP-based phylogenetic tree of 119 NTS from this study was constructed as previously described ^2^. Their genomic sequences were mapped to a reference sequence of *Salmonella* Typhimurium SL1344 by Snippy v4.4.4 (https://github.com/tseemann/snippy). A maximum-likelihood phylogenetic tree with 1,000 bootstraps was generated using IQ-TREE v1.6.12 ^3^. Besides, as an emerging *Salmonella* serovar associated with human infections in China, and further accounting for the leading proportion of invasive cases in our study, One Health scale genomic tracking for *S.* Goldcoast was performed. To investigate the genomic relatedness of seven *S.* Goldcoast isolates from this study and *S.* Goldcoast isolates with global context (i.e., 255 Chinese isolates and 65 global isolates, **Supplementary Dataset 1**), a core-genome SNP-based phylogenetic tree was built using the Harvest suite ^4^. The recombination regions of genomic sequences were removed, and pairwise SNPs were calculated by Parsnp ^5^. The phylogenetic tree and associated data were visualized with iTOL v6.7.3.

**Statistical analysis**

For non-normally distributed continuous variables, the data was shown as medians and interquartile ranges (IQRs). For categorical variables, the data were presented as percentages. The comparison between iNTS and non-iNTS infections was calculated with the Mann-Whitney test for non-normally distributed continuous variables, the t-test for normally distributed continuous variables, and the Chi-square or Fisher’s exact test for categorical variables. All statistical analyses were performed using SPSS 26.0 software (IBM, Armonk, NY, USA) or GraphPad Prism 9.5 software (GraphPad Software Inc., CA, USA). *P* <0.05 was considered statistically significant.

**References**

1 Ke Y, Lu W, Liu W, Zhu P, Chen Q, Zhu Z. Non-typhoidal *Salmonella* infections among children in a tertiary hospital in Ningbo, Zhejiang, China, 2012-2019. *PLoS Negl Trop Dis* 2020; 14(10): e0008732.

2 Li Y, Teng L, Xu X, Li X, Peng X, Zhou X, et al. A nontyphoidal *Salmonella* serovar domestication accompanying enhanced niche adaptation. *EMBO Mol Med* 2022; 14(11): e16366.

3 Xu X, Chen Y, Pan H, Pang Z, Li F, Peng X, et al. Genomic characterization of *Salmonella* Uzaramo for human invasive infection. *Microb Genom* 2020; 6(7).

4 Teng L, Zou G, Zhou Y, Li J, Song Z, Dong X, et al. Phage controlling method against novel freshwater-derived Vibrio parahaemolyticus in ready-to-eat crayfish (Procambarus clarkii). *Food Res Int* 2022; 162(Pt A): 111986.

5 Hu B, Hou P, Teng L, Miao S, Zhao L, Ji S, et al. Genomic Investigation Reveals a Community Typhoid Outbreak Caused by Contaminated Drinking Water in China, 2016. *Front Med (Lausanne)* 2022; 9: 753085.

**
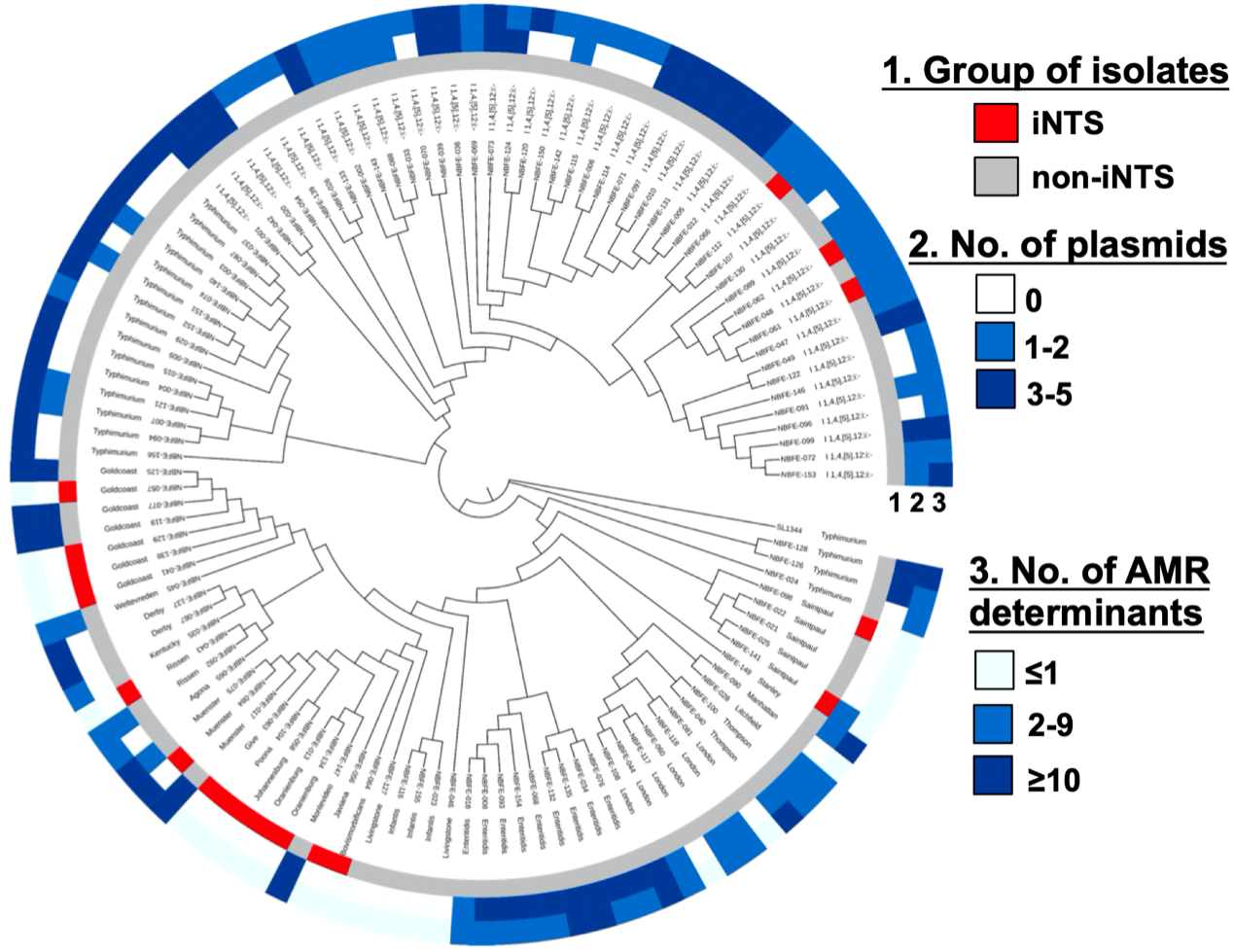
**

**Figure S1.** Phylogenetic tree of 119 NTS isolates. The phylogenomic tree was constructed using the core genome-based SNPs of *Salmonella* genomes, with *S.* Typhimurium SL1344 as a reference genome. There rings from inter-layer to outer-later indicate group of isolates, number of plasmids, and number of AMR determinants, respectively.

**
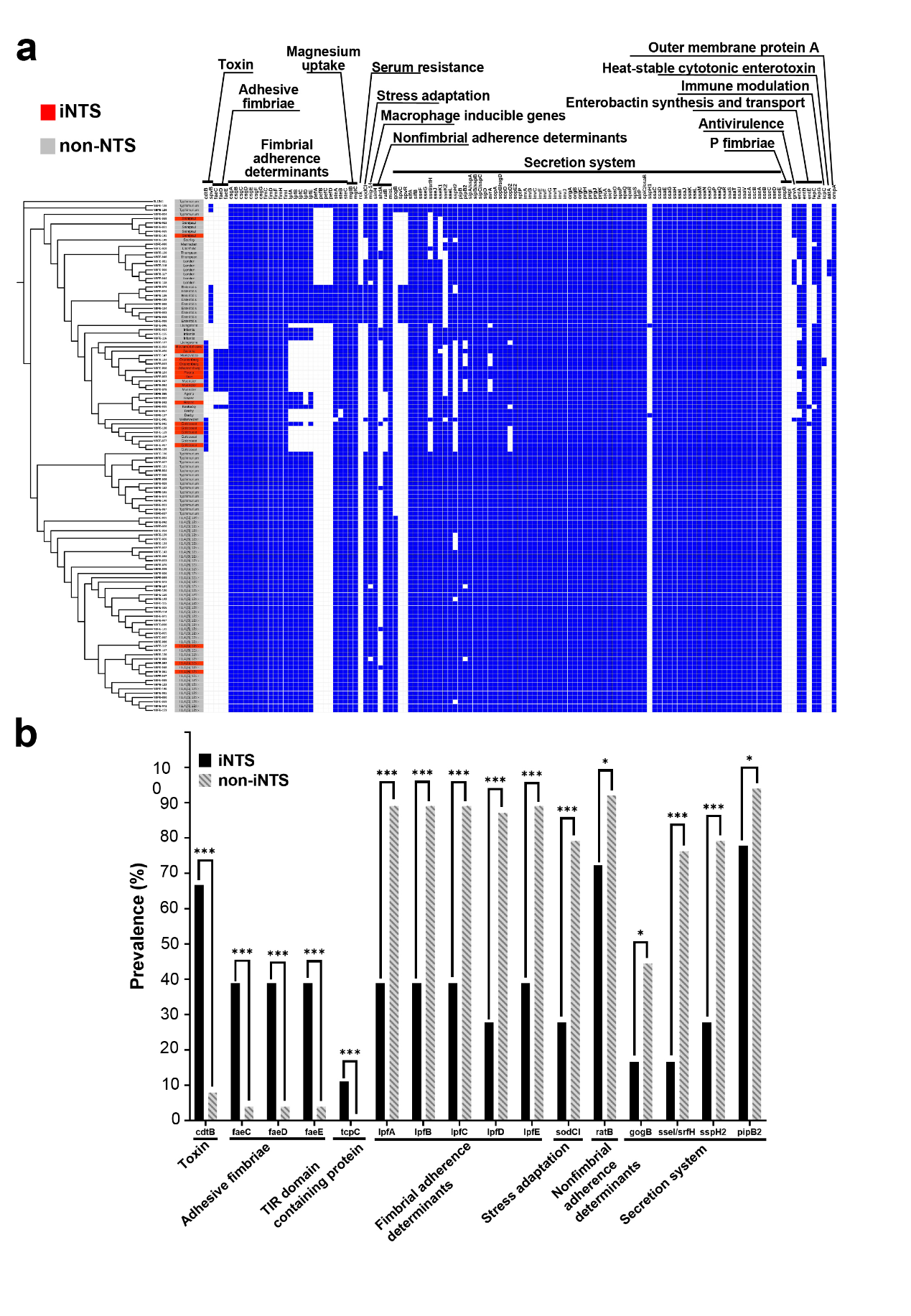
**

**Figure S2**. Characterization of virulence factors in iNTS and non-iNTS isolates. (a) Phylogenetic tree and virulence genes in NTS isolates. The presence (blue) and absence (white) of virulence genes are indicated by different colours. The functions of virulence genes are label on the top of the gene names. (b) Prevalence of virulence genes between iNTS and non-iNTS groups. Statistical analysis is conducted using Chi-square or Fisher’s exact test. *** (*P* < 0.001); ** (*P* < 0.01); * (*P* < 0.05); ns (no significant difference).

**Supplementary Figures**

**
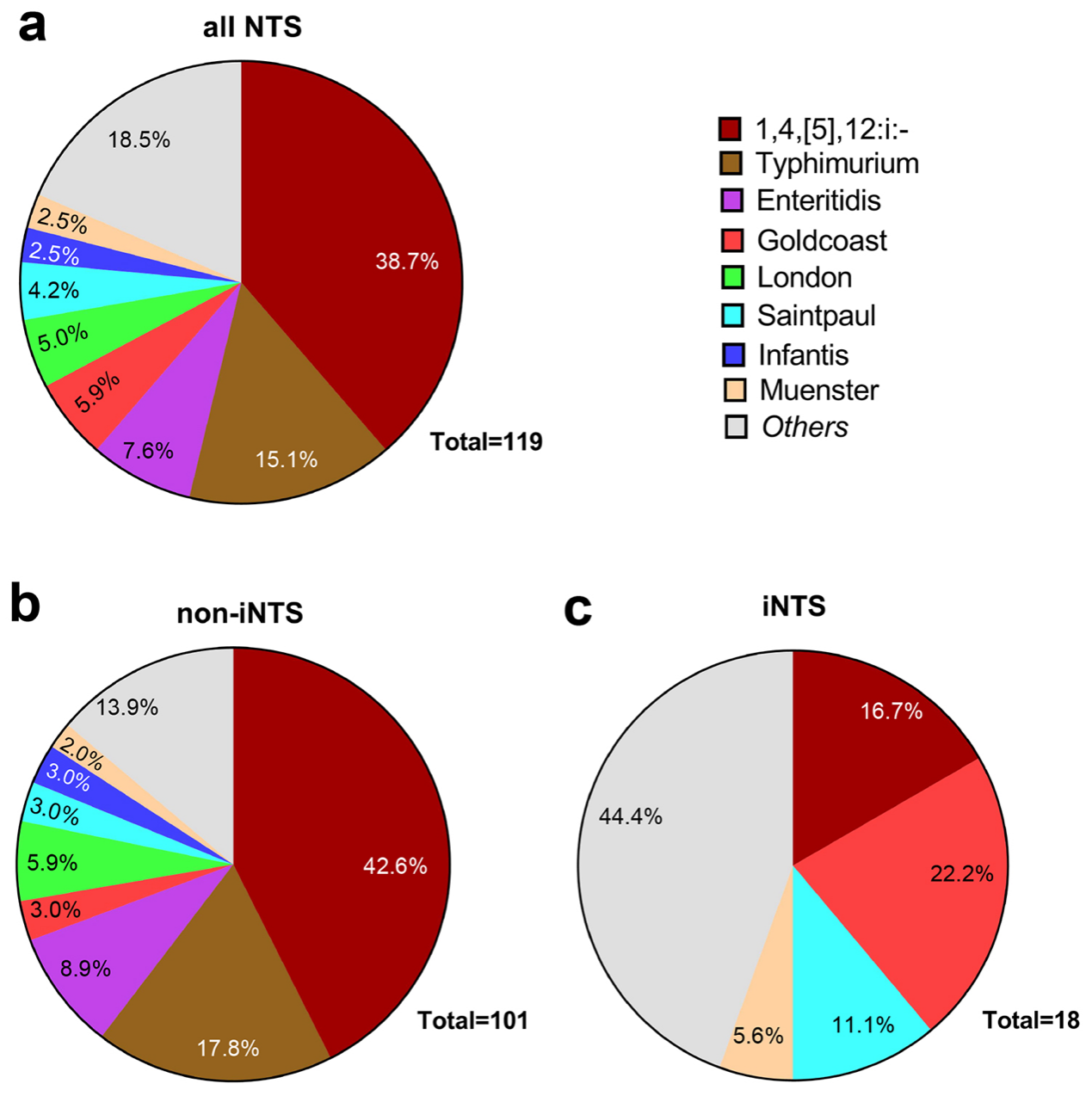
**

**Figure S3. Proportion of NTS isolates by serovar.** (a) The predominant serovar of all NTS isolates. (b) The major non-iNTS serovars; (c) The percentage of iNTS isolates by serovar.

**
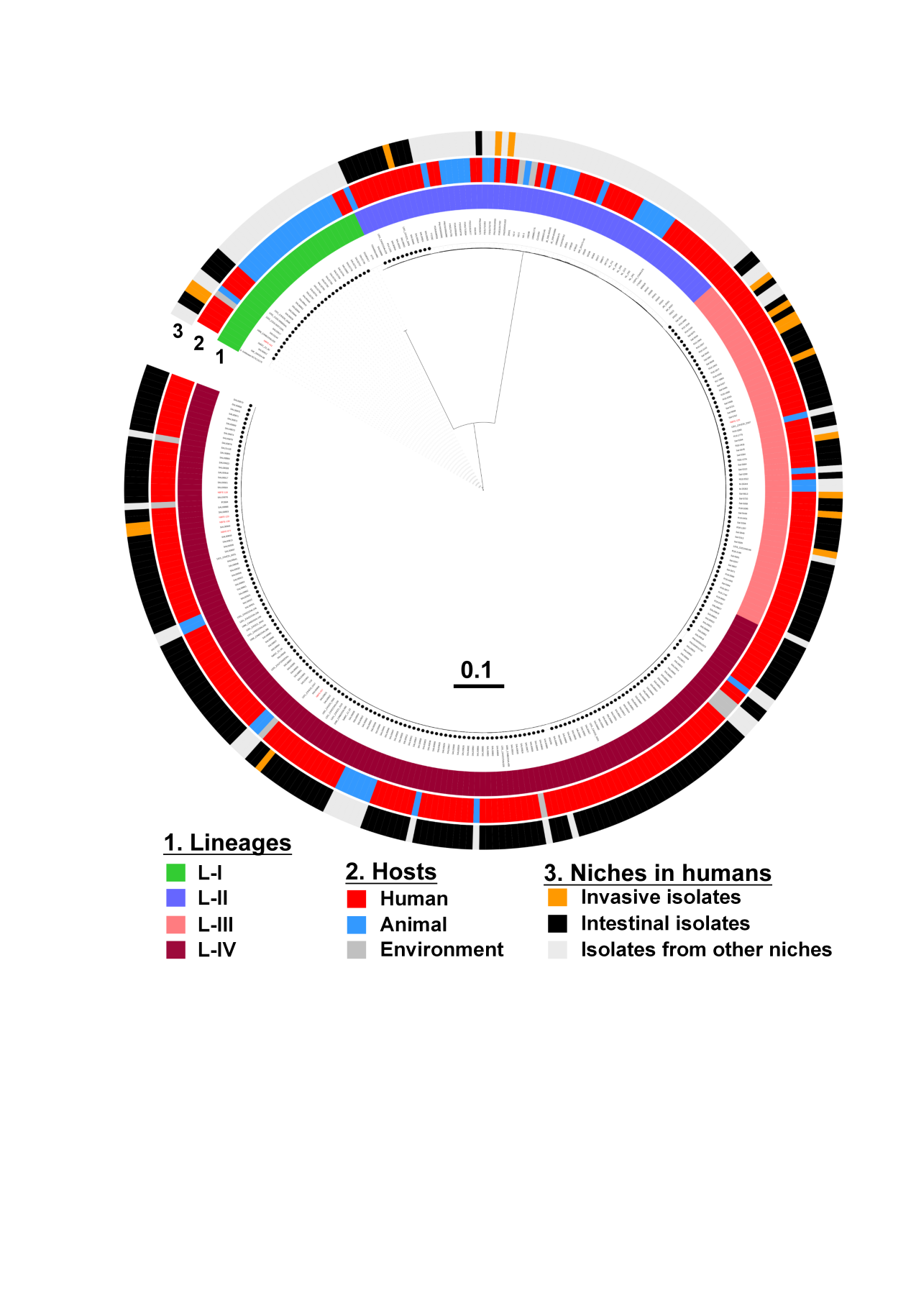
**

**Figure S4.** Phylogenetic tree of global *Salmonella* Goldcoast isolates. The tree was constructed using the core genome-based SNPs of 327 *Salmonella* genomes, including 7 genomes from the current study (strain names in red), with genomic sequence of *Salmonella* Goldcoast NCTC 13175 as a reference. Isolates with black dots represents the isolates from China.

**Supplementary Tables**

**Table S1.** A list of clinical isolates with pediatric infections used in this study. See excel sheet.

**Table S2.** Demographic and clinical manifestations of 119 children with NTS infections.

| **Characteristic** | **Total**  **(n=119)** | **iNTS infections**  **(n=18)** | **Non-iNTS infections**  **(n=101)** | ***P*** |
| --- | --- | --- | --- | --- |
| **Gender** |  |  |  |  |
| Male | 63 (52.9%) | 11 (61.1%) | 52 (51.5%) | 0.451 |
| Female | 56 (47.1%) | 7 (38.9%) | 49 (48.5%) |  |
| **Age** |  |  |  |  |
| Age below 5 years | 110 (92.4%) | 18 (100%) | 92 (91.1%) | 0.405 |
| Age (month) (IQR) | 13 (10-20) | 15 (12-20) | 13 (9-24) | 0.504 |
| **Symptoms at admission** |  |  |  |  |
| Fever | 109 (91.6%) | 17 (94.4%) | 92 (91.1%) | 0.991 |
| Convulsions | 11 (9.2%) | 0 (0.0%) | 11 (10.9%) | 0.304 |
| Vomiting | 29 (24.4%) | 1 (5.6%) | 28 (27.7%) | 0.085 |
| Diarrhea | 112 (94.1%) | 11 (61.1%) | 101 (100%) | <0.001 |
| Mucus in stool | 71 (59.7%) | 6 (33.3%) | 65 (64.4%) | 0.013 |
| Blood in stool | 100 (84.0%) | 13 (72.2%) | 87 (86.1%) | 0.256 |
| **Laboratory examination results^*^** |  |  |  |  |
| WBCs (×10^9/L) (IQR) | 9.6  (7.2-12.7) | 10.6  (8.4-12.4) | 9.4  (7.1-12.7) | 0.448 |
| CRP (mg/L) (IQR) | 28.1  (10.4-79.3) | 22.5  (9.9-48.5) | 29.3  (10.4-88.4) | 0.470 |
| PCT (ng/mL) (IQR) | 0.3  (0.1-0.9) | 0.4  (0.2-0.6) | 0.3  (0.1-1.0) | 0.667 |
| HB (g/dL) (mean ±SD) | 11.7 ±1.1 | 11.4 ±0.7 | 11.8 ±1.2 | 0.183 |

*Seventeen children’s results of WBCs, CRP, PCT and HB in iNTS infection cases were available, 100 children’s results of WBCs, CRP, and HB and 96 children’s results of PCT in non-iNTS infection cases were available.

WBCs: white blood cell counts, CRP: C-reactive protein, PCT: procalcitonin, HB: Haemoglobin. iNTS: invasive nontyphoidal *Salmonella*.

**Table S3.** The correlation of phenotypic and genotypic resistance.

|  |  | **No. of isolates** | | | |  |  |
| --- | --- | --- | --- | --- | --- | --- | --- |
|  |  | **Resistant phenotype** | | **Susceptible phenotype** | |  |  |
| **Antimicrobial class** | **Antimicrobials** | **Resistant genotype (TP)** | **Susceptible genotype (FN)** | **Resistant genotype (FP)** | **Susceptible genotype (TN)** | **Sensitivity (%)** | **Specificity (%)** |
| **Beta-lactam** | AMP | 81 | 3 | 3 | 32 | 96.4 | 91.4 |
|  | CAZ | 24 | 2 | 13 | 80 | 92.3 | 86.0 |
|  | ATM | 30 | 5 | 4 | 80 | 85.7 | 95.2 |
|  | FEP | 26 | 3 | 10 | 80 | 89.7 | 88.9 |
|  | CRO | 30 | 9 | 1 | 79 | 76.9 | 98.8 |
|  | IPM | 1 | 0 | 0 | 118 | 100.0 | 100.0 |
|  | ETP | 1 | 4 | 0 | 114 | 20.0 | 100.0 |
| **Trimethoprim** | SXT | 41 | 2 | 7 | 69 | 95.3 | 90.8 |
| **Quinolones or Mutain** | LEV | 66 | 5 | 1 | 47 | 93.0 | 97.9 |
|  | CIP | 50 | 4 | 17 | 48 | 92.6 | 73.8 |
| **Total** |  | 350 | 37 | 56 | 747 | 90.4% | 93.0% |

Abbreviation for antimicrobials: Ertapenem (ETP), Imipenem (IPM), Levofloxacin (LEV), Ciprofloxacin (CIP), Aztreonam (ATM), Cefepime (FEP), Ceftriaxone (CRO), Ceftazidime (CAZ), Trimethoprim-sulfamethoxazole (TMP-SMX), and Ampicillin (AMP).

**Table S4.** A list of antimicrobial resistance genes and phenotypic resistance towards examined antimicrobials.

| **Antimicrobial resistance genes** | **Phenotypic resistance** |
| --- | --- |
| **Beta-lactam** |  |
| *bla*_CMY-2_ | AMP; CAZ; TZP; SAM |
| *bla*_CTX-M-130_ | AMP; ATM; FEP; CAZ; CRO; |
| *bla*_CTX-M-14_ | AMP; ATM; FEP; CAZ; CRO; |
| *bla*_CTX-M-14b_ | AMP; ATM; FEP; CAZ; CRO; |
| *bla*_CTX-M-55_ | AMP; ATM; FEP; CAZ; CRO; |
| *bla*_CTX-M-64_ | AMP; ATM; FEP; CAZ; CRO; |
| *bla*_CTX-M-65_ | AMP; ATM; FEP; CAZ; CRO; |
| *bla*_DHA-1_ | AMP; CAZ; TZP; SAM |
| *bla*_DHA-15_ | AMP; CAZ; TZP; SAM |
| *bla*_NDM-5_ | AMP; FEP; CAZ; ETP; IPM; TZP; SAM |
| *bla*_OXA-10_ | AMP; ATM; TZP; SAM |
| *bla*_OXA-1_ | AMP; FEP; TZP; SAM |
| *bla*_TEM-141_ | AMP; |
| *bla*_TEM-1A_ | AMP; |
| *bla*_TEM-1B_ | AMP; |
| **Trimethoprim** |  |
| *dfrA12* | SXT |
| *dfrA14* | SXT |
| *dfrA17* | SXT |
| *dfrA27* | SXT |
| **Quinolones** |  |
| *oqxA* | LEV; CIP |
| *oqxB* | LEV; CIP |
| *qnrB4* | LEV; CIP |
| *qnrB6* | LEV; CIP |
| *qnrS1* | LEV; CIP |
| *qnrS2* | LEV; CIP |
| *gyrA* | LEV; CIP |

**Supplemental Dataset 1.** A detailed list of 327 *Salmonella* Goldcoast isolates in this study. See excel sheet
